# Effects of Cervical Motion Restriction on Wheelchair Propulsion Velocity and Upper-Limb Kinematics in Healthy Individuals

**DOI:** 10.3390/s26185829

**Published:** 2026-09-14

**Authors:** Tomoe Sobu, Yusuke Sekiguchi, Ziheng Wang, Keita Honda, Kaoru Kuroki, Naoki Suzuki, Ryoichi Nagatomi, Satoru Ebihara

**Affiliations:** 1 Department of Rehabilitation Medicine, Tohoku University Graduate School of Medicine, Sendai 980-8574, Japan; honda.kt@kumamoto-hsu.ac.jp (K.H.); naoki.suzuki.e3@tohoku.ac.jp (N.S.); satoru.ebihara.c4@tohoku.ac.jp (S.E.); 2 Department of Rehabilitation, Faculty of Health Sciences, Tohoku Fukushi University, Sendai 981-8522, Japan; kuroki@tfu.ac.jp; 3 Research Management Center, Organization for Research Promotion, Tohoku University, Sendai 980-8577, Japan; yusuke.sekiguchi.b2@tohoku.ac.jp; 4 Graduate School of Dentistry, Tohoku University, Sendai 980-8575, Japan; 5 Designing Future Health Initiative, Center for Promotion of Innovation Strategy, Tohoku University, Sendai 980-8576, Japan; wang.ziheng.a3@tohoku.ac.jp (Z.W.); ryoichi.nagatomi.c4@tohoku.ac.jp (R.N.); 6 Graduate School of Health Sciences, Kumamoto Health Science University, Kumamoto 861-5598, Japan; 7 Open Innovation Strategy Organization, Tohoku University, Sendai 980-8577, Japan; 8 Department of Frailty Prevention and Metabolic Regulation, Healthspan Research Center (HeSReC), Tohoku University, Sendai 980-8575, Japan

**Keywords:** wheelchair propulsion, kinematics, cervical fixation, wheelchair rugby, velocity increment

## Abstract

Forward head flexion during wheelchair propulsion has been suggested to compensate for reduced trunk muscle strength in individuals with spinal cord injury, but the influence of restricting head–neck movement on propulsion remains unclear. This study examined wheelchair propulsion under cervical fixation and non-fixation conditions. Fourteen healthy young men (20.7 ± 0.5 years) performed 5 m wheelchair propulsion under both conditions. Propulsion was recorded with a smartphone camera, and coordinates of the left ear, shoulder, elbow, and wrist were extracted using You Only Look Once version 7 (YOLO v7) to calculate upper-arm segment and elbow angles and angular accelerations. Analysis focused on the first three propulsion cycles after movement onset. Propulsion velocity showed a significant main effect of measurement point, but neither the main effect of cervical fixation nor the condition-by-point interaction was significant. Velocity increment differed significantly among measurement intervals, whereas the condition effect and interaction were not significant. Cervical fixation was associated with a smaller minimum upper-arm segment angle and greater upper-arm segment excursion, maximum elbow angle, and elbow range of motion. A significant condition-by-cycle interaction was found for minimum elbow angle, with a smaller angle under fixation during the first cycle. Minimum upper-arm segment angular acceleration in the flexion direction also differed among cycles. Thus, cervical fixation was associated with changes in upper-limb kinematics, but a statistically significant change in propulsion velocity was not demonstrated. These preliminary findings provide a basis for future studies in wheelchair users and athletes.

## 1. Introduction

Wheelchair rugby was developed in Canada in 1977 for athletes with tetraplegia who required an alternative to wheelchair basketball [1]. Since then, it has evolved into an international Paralympic sport characterized by frequent wheelchair contact, specialized equipment, and participation by athletes with a wide range of eligible impairments [2,3]. In addition to its competitive nature, regular wheelchair rugby training has been associated with improved pulmonary function and functional ability in people with tetraplegia [4,5]. In a cross-sectional study of competitive male wheelchair rugby athletes with tetraplegia, practicing at least twice per week was associated with lower depressive symptoms and perceived stress [6]. At the same time, the sport relies on a classification system intended to minimize the impact of impairment on competition outcomes [7]. Because class assignment combines upper-extremity and trunk evaluation scores, athletes within the same sport class may have different activity profiles [3]. Wheelchair setup can also affect propulsion kinematics and sprint performance, and the effects vary across athletes [8]. Moreover, the evidence base supporting the validity and reliability of field tests used to assess physical condition in wheelchair rugby remains in its infancy [9]. These issues highlight the need for objective biomechanical indicators that are directly related to wheelchair rugby performance.

Initial push-rim mechanical outputs have shown large associations with 3, 5, and 12 m sprint performance in elite wheelchair rugby players [9]. Across elite wheelchair athletes from several disciplines, propulsion technique and power output varied with functional ability, while weekly training hours showed small associations with peak power, peak torque, work per push, and total force [10]. Previous studies have shown that individuals with upper-limb impairments exhibit higher stroke frequency, shorter hand–rim contact time, smaller joint excursions at the wrist, elbow, and shoulder, and lower propulsive and compressive joint forces than individuals without upper-limb impairment [11]. Mechanical analysis has further indicated that the shoulder is predominantly in a propulsion configuration, although close to stabilization, whereas the elbow and wrist are mainly in stabilization configurations during manual wheelchair propulsion [12]. In addition, elbow translation, rather than elbow angle, has been associated with propulsion speed in individuals with tetraplegia [13]. Together, these findings indicate that upper-limb kinematics are closely related to propulsion performance, but they do not fully explain how athletes compensate for impaired trunk function.

Trunk function is also an important contributor to wheelchair propulsion. Sensorimotor trunk impairments, together with their effects on postural stability and upper-limb strength-generating capacity, may reduce the capacity of individuals with a spinal cord injury to propel a manual wheelchair efficiently [14]. Among manual wheelchair users with tetraplegia, the ranges of neck and upper-trunk forward flexion increased as propulsion speed increased, whereas lateral flexion and axial rotation did not significantly increase; these movements were interpreted as compensation for trunk muscle weakness [15]. This suggests that forward flexion of the head and trunk may serve as a compensatory strategy for reduced trunk muscle function during propulsion. However, the specific contribution of cervical motion to propulsion performance has not been examined in detail, and the mechanism by which cervical motion influences wheelchair propulsion remains unclear. As an initial step, healthy participants were examined in the present study to evaluate the effects of cervical motion restriction on upper-limb kinematics during wheelchair propulsion under controlled conditions. In individuals with tetraplegia or wheelchair rugby athletes, impairment level, residual muscle strength, trunk control, spasticity, wheelchair experience, and training history may vary substantially and may confound the interpretation of cervical and upper-limb coordination. Therefore, this study used healthy participants to examine the biomechanical relationship between cervical restriction and propulsion performance under controlled conditions.

Clarifying how a cervical fixation condition affects wheelchair propulsion may provide a biomechanical basis for future research on coaching and classification in wheelchair rugby. Therefore, the purpose of this study was to examine the influence of a cervical fixation condition on wheelchair propulsion velocity and upper-limb kinematics by comparing wheelchair propulsion under cervical fixation and non-fixation conditions in healthy young men.

## 2. Materials and Methods

### 2.1. Study Design and Participants

This single-center experimental study with a repeated-measures design was conducted at Tohoku University School of Medicine, Sendai, Japan, and was approved by the Ethics Committee of the Graduate School of Medicine, Tohoku University (protocol code: 2024-1-416). The study was carried out from September to December 2024.

Twenty-two healthy young men underwent testing. Eight participants were excluded because fewer than three valid observations were available for at least one combination of experimental condition and propulsion cycle. Therefore, 14 participants were included in the final analysis (mean age, 20.7 ± 0.5 years). Eligible participants were men aged 20 to 60 years. Individuals with orthopedic or neurological disorders that could affect the measurements were excluded. None of the included participants used a wheelchair habitually or had experience in wheelchair sports. They had limited prior exposure to wheelchair operation through classes or practical training, but the amount of experience was not recorded. The study procedures were explained to all participants before testing, and written informed consent was obtained from all participants. Height, body weight, body mass index (BMI), upper arm length, forearm length, hand length, and grip strength were measured for all participants (Table 1).

### 2.2. Experimental Procedures

**Figure 1:**
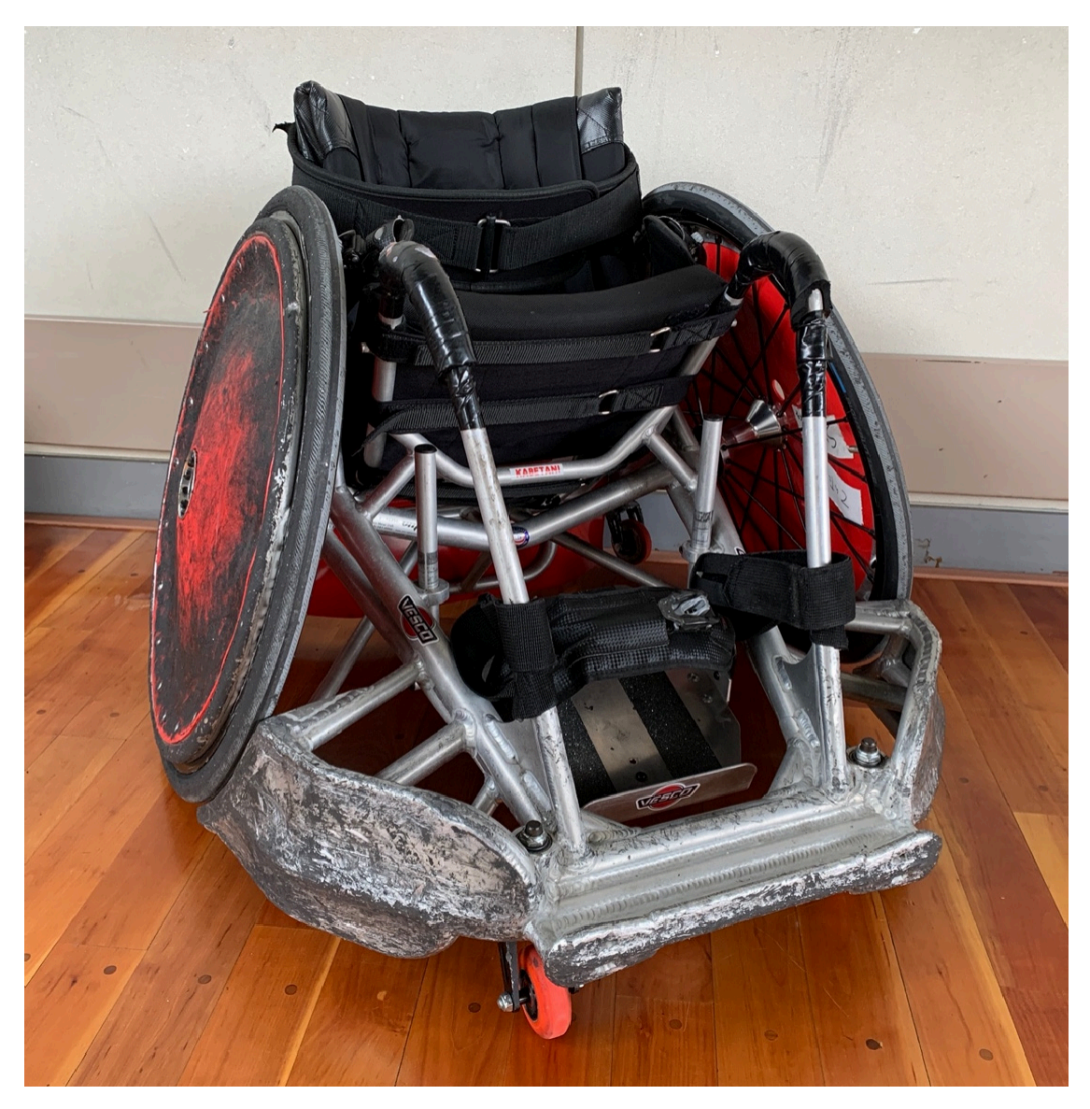
Competition wheelchair for wheelchair rugby. The experimental measurements were conducted using a wheelchair manufactured by VESCO Metal Craft (Chula Vista, CA, USA).

All measurements were performed in an indoor laboratory at Tohoku Fukushi University. The same wheelchair rugby wheelchair manufactured by VESCO Metal Craft (Chula Vista, CA, USA) was used for all participants and conditions (Figure 1). The wheelchair configuration was not adjusted for participant body size and was kept unchanged between the cervical fixation and non-fixation conditions. The wheelchair was equipped with belts to secure the trunk, pelvis, knees, and feet during testing. Tire pressure was set at 8.0 kgf/cm^2^ (approximately 785 kPa) for all participants and both conditions. It was rechecked when measurement irregularities prompted confirmation, but was not measured routinely before every participant. Participants were instructed to sit with the pelvis and buttocks against the rear of the seat, keep both feet on the footrests, and place both hands at the top of the drive wheels at a position from which they could apply force comfortably. The same seating setup was used in both conditions. Before data collection, participants completed approximately 10 min of warm-up and familiarization while seated in the wheelchair.

**Figure 2:**
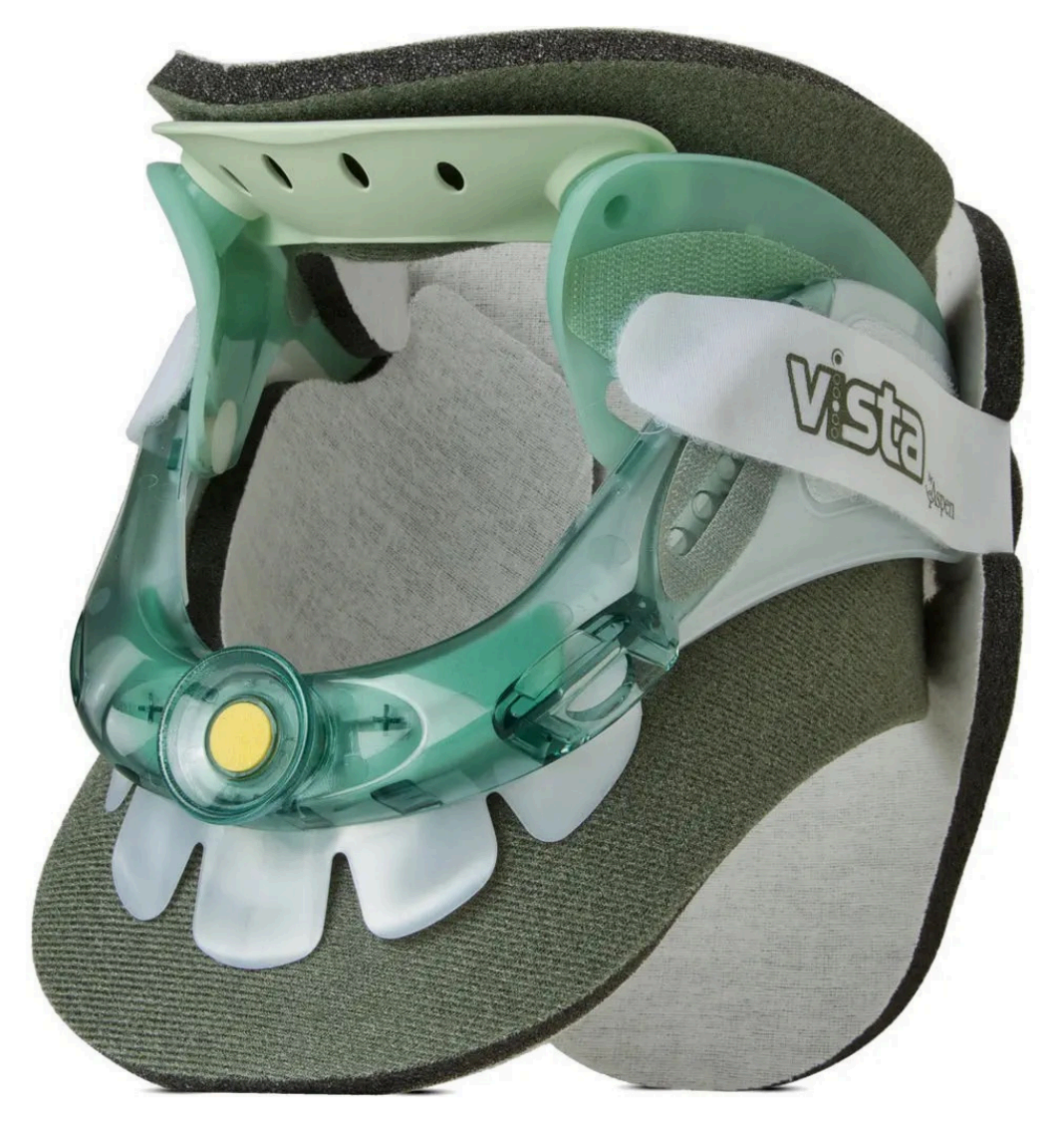
Cervical collar. Cervical fixation during the measurements was achieved using a cervical collar (Vista^®^ TX, Aspen Medical Products).

**Figure 3:**
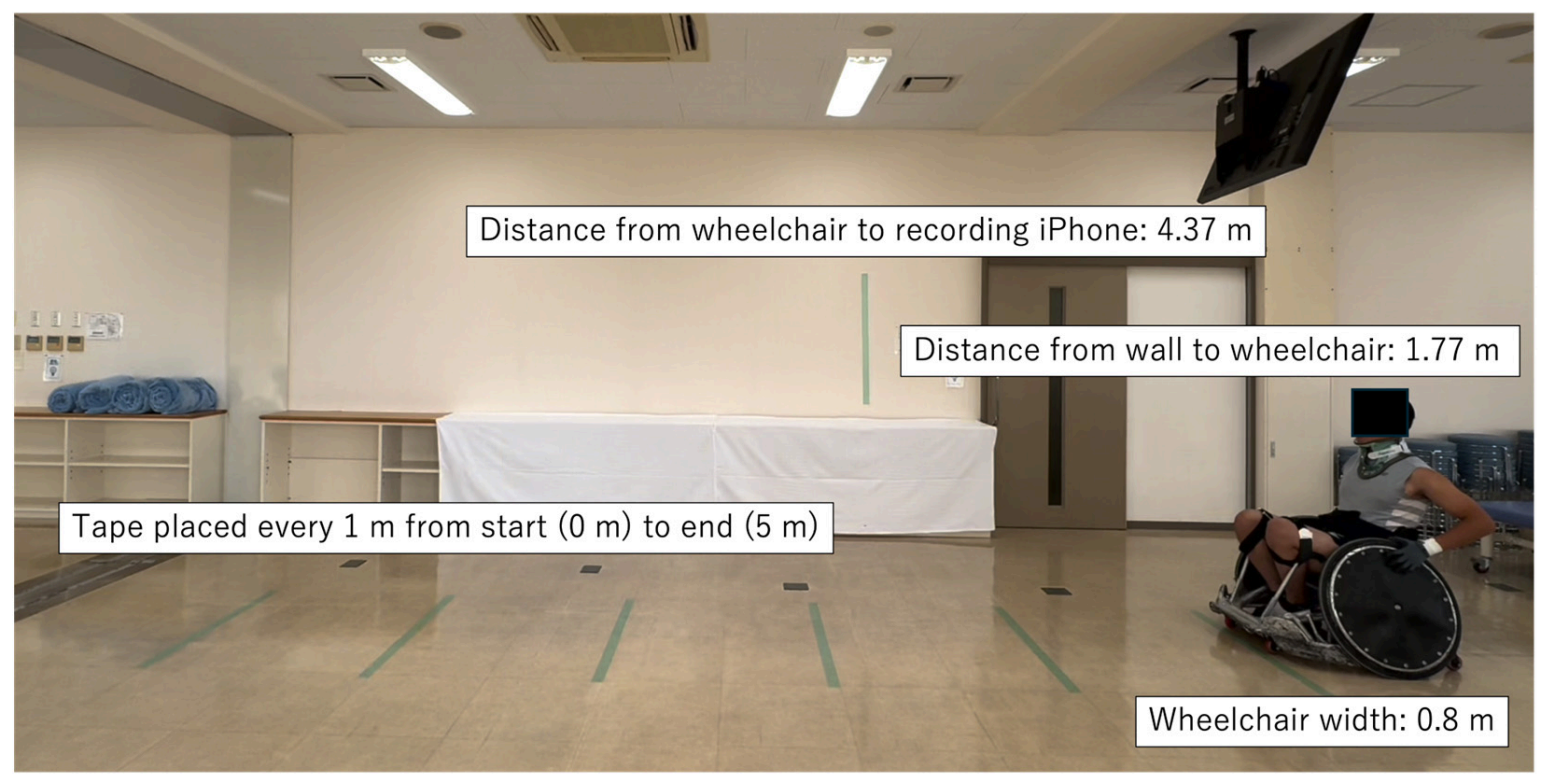
Experimental setting for wheelchair propulsion. All measurements took place in an indoor laboratory. The 5 m linear path featured markers at 1 m increments to ensure accurate distance calibration during the subsequent analysis.

Wheelchair propulsion was assessed under two conditions: with cervical fixation using a rigid cervical collar (Vista^®^ TX; Aspen Medical Products, Irvine, CA, USA) and without cervical fixation (Figure 2). The collar height and fit were adjusted individually and fitted by the same investigator using the manufacturer’s recommended procedure. Its position was checked to support the mandible. Tightness was adjusted based on the participant’s report of no breathing discomfort and a subjective sense of fixation, and fit and symptoms were checked again after application. No predefined quantitative criterion was used to standardize tightness. The experimental course consisted of a straight 5 m path. Under each condition, participants performed a minimum of five and a maximum of 10 trials. During data collection, trials affected by obvious measurement failure, hand slippage during propulsion, or other events that prevented valid recording were discarded, and testing was continued until five trials judged valid at the time of measurement had been obtained, with a maximum of 10 attempts per condition. Consequently, the number of attempts varied among participants and conditions. Perceived fatigue was checked after each trial, and a rest period of approximately 2 to 3 min was provided when necessary. Tape markers were placed at 1 m intervals along the course to allow distance calibration during video analysis (Figure 3). The order of the cervical fixation and non-fixation conditions was randomized separately for each participant using random numbers generated in Microsoft Excel (Microsoft Corp., Redmond, WA, USA).

### 2.3. Video Acquisition

**Figure 4:**
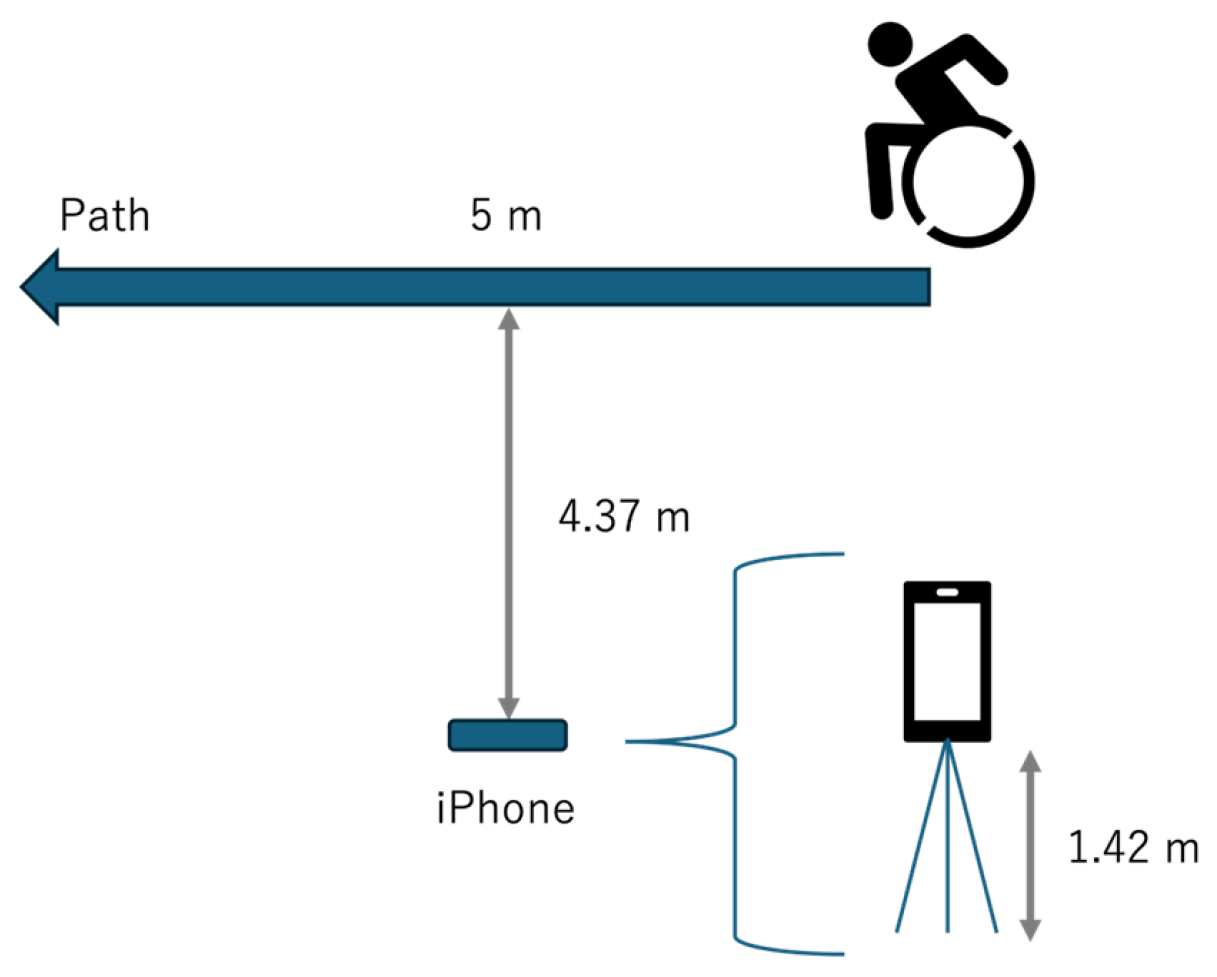
Camera setup. The movements during wheelchair propulsion were recorded in the sagittal plane from the left side using a single smartphone camera (iPhone 15 Pro; Apple Inc.). The camera was mounted on a tripod at a height of 1.42 m. The distance from the wall to the subject was 1.77 m, and the distance from the subject to the camera was 4.37 m.

Wheelchair propulsion was recorded in the sagittal plane from the participant’s left side using the built-in camera of a smartphone (iPhone 15 Pro; Apple Inc., Cupertino, CA, USA) at 30 Hz. Videos were recorded at a resolution of 1920 × 1080 pixels. The camera was mounted on a tripod at a height of 1.42 m. The distance from the wall to the participant was 1.77 m, and the distance from the participant to the camera was 4.37 m (Figure 4).

### 2.4. Data Analysis

#### 2.4.1. Wheelchair Propulsion Velocity and Velocity Increment

Wheelchair propulsion velocity was calculated from videos recorded at 30 frames/s. A single examiner, who was also the principal investigator, visually identified the frame in which the front bumper crossed each marker at 0, 1, 2, 3, 4, and 5 m. Videos were reviewed multiple times during frame identification. The examiner was not blinded to condition, and neither intra-rater nor inter-rater reliability was formally quantified. For marker distance d, the cumulative passage time from movement onset was calculated as t_d = f_d/30, where f_d was the elapsed frame count, and cumulative average propulsion velocity was calculated as v_d = d/t_d = 30d/f_d. Velocity increment for each interval was calculated as the difference between adjacent cumulative velocity values (Δv_d = v_d − v_(d − 1), with v_0 = 0); it was not treated as acceleration because it was not divided by elapsed time. The temporal resolution was 1/30 s (approximately 0.033 s). Across the observed mean velocities, a one-frame shift in marker-crossing identification corresponded to an estimated velocity difference of approximately 0.03–0.05 m/s, depending on the measurement point.

#### 2.4.2. Extraction of Two-Dimensional Coordinates

**Figure 5:**
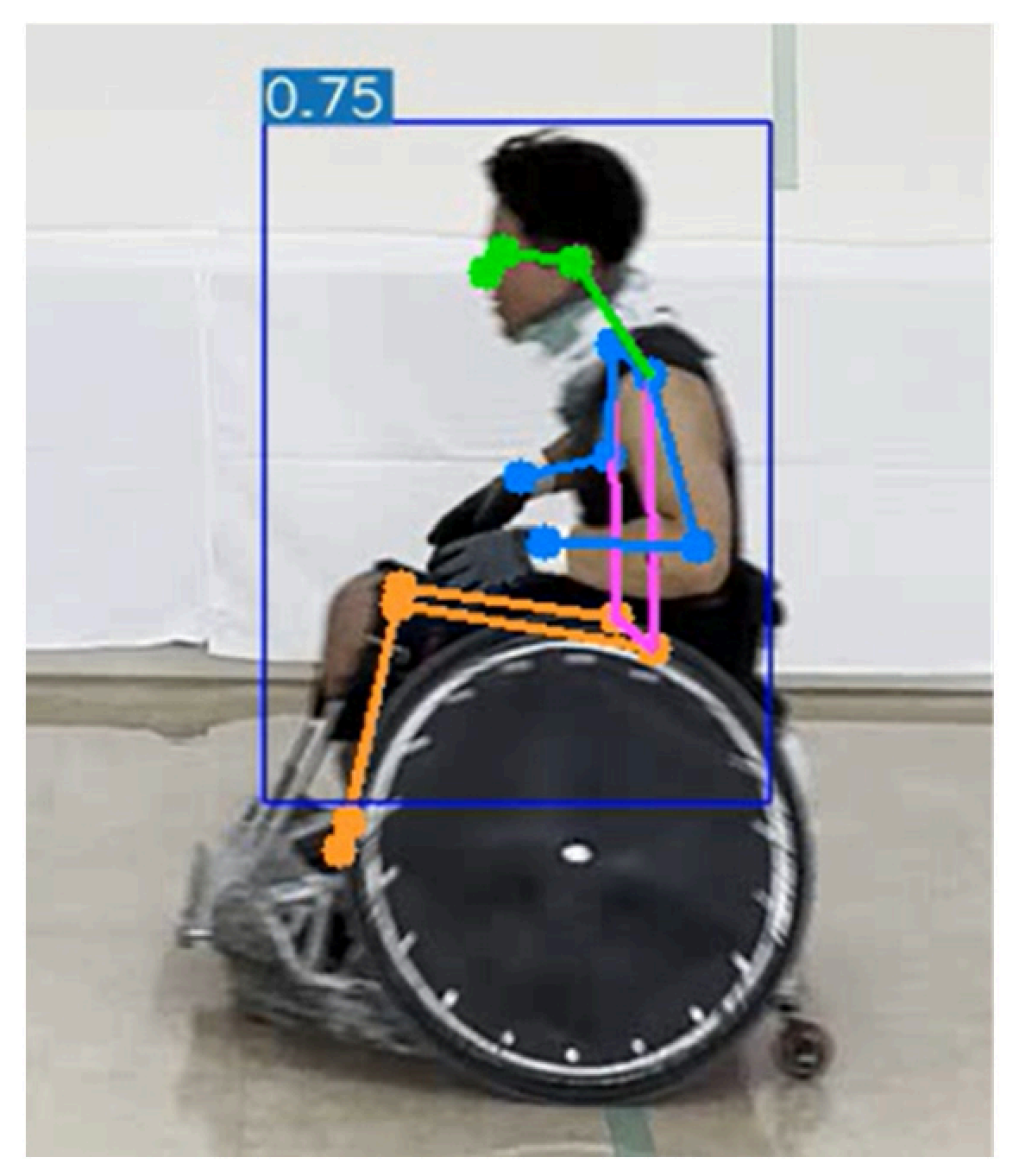
Pose estimation via image analysis. Colored markers and lines indicate the body keypoints and skeletal connections estimated using the YOLOv7 algorithm. The blue bounding box indicates the detected participant, and the displayed value represents the detection confidence score. Specifically, the coordinates for the left ear, left shoulder joint, left elbow joint, and left wrist joint were extracted for the analysis.

Two-dimensional body coordinates during wheelchair propulsion were estimated from the recorded videos using You Only Look Once version 7 (YOLO v7). The archived coordinate files contained x- and y-coordinates for 17 keypoints in the COCO format; confidence scores were not retained. Detections located in a restricted upper-right region of the image and corresponding to a person other than the target participant were excluded on the basis of coordinate position. The exact coordinate thresholds, model weights, implementation version, confidence threshold, interpolation procedure, and original execution environment could not be retrospectively verified from the archived materials. For the present analysis, the x- and y-coordinates of the left ear, left shoulder, left elbow, and left wrist were used (Figure 5). No study-specific manual annotation or positional or angular accuracy validation data were available.

#### 2.4.3. Joint Angles and Angular Accelerations

**Figure 6:**
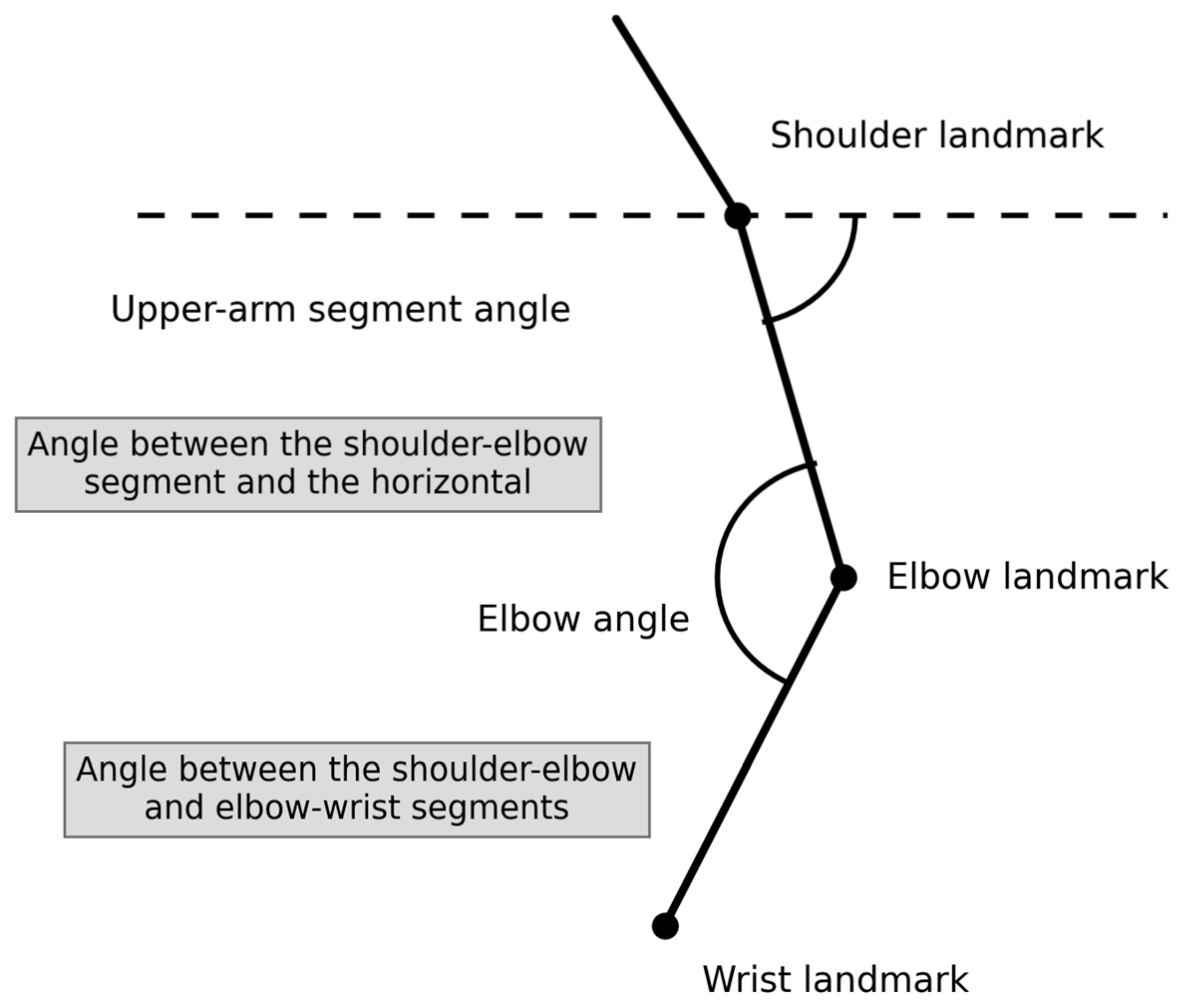
Definition of kinematic angles. The upper-arm segment angle is defined as the angle between the horizontal axis and the shoulder–elbow segment. The elbow angle is defined as the angle formed by the shoulder–elbow and elbow–wrist segments.

Elbow joint angle and upper-arm segment angle were calculated by vector operations using the extracted coordinates. The elbow angle was calculated as the included angle between the vectors from the left elbow to the left shoulder and from the left elbow to the left wrist. The upper-arm segment angle was calculated as the angle between the vector from the left shoulder to the left elbow and the leftward horizontal axis. It therefore represents segment orientation relative to the image horizontal axis and should not be interpreted as an anatomical shoulder joint angle relative to the trunk (Figure 6).

The raw angle time series were smoothed using a one-dimensional Gaussian filter with sigma = 2 samples (two frames). Angular velocity and angular acceleration were then calculated as the first and second numerical differences, respectively, of the Gaussian-smoothed angle series. The first differences were multiplied by the sampling rate (30 Hz) and the second differences by its square (900) to express angular velocity in °/s and angular acceleration in °/s^2^. For the upper-arm segment and elbow angles, larger values indicated extension and smaller values indicated flexion; for angular velocity and angular acceleration, positive values indicated the extension direction, whereas negative values indicated the flexion direction.

Based on the processed time-series data, kinematic features were extracted for each propulsion cycle. One propulsion cycle was defined as the interval from one point of maximal upper-arm segment extension to the next. For the upper-arm segment and elbow angles, the maximum and minimum values were extracted, and their difference was defined as segment excursion or elbow range of motion, respectively. For angular acceleration, the maximum and minimum values were extracted. Because the elbow angular-acceleration waveform showed a bimodal pattern, two maxima were extracted for the elbow, one from the first half of the cycle and one from the second half. In both angle and angular acceleration data, an increase indicated extension, whereas a decrease indicated flexion.

Preliminary analysis showed that velocity increments in the initial 2 m were greater than those in the subsequent intervals. The first three propulsion cycles generally corresponded to this initial section, with the third cycle occurring near the 2 m point. Therefore, the first through third propulsion cycles after movement onset were selected for analysis. Kinematic observations were reviewed separately for each condition and propulsion cycle. Observations affected by measurement failure were excluded. During offline inspection, the same investigator who recorded the data excluded 15 observations identified in the archived worksheet as extreme numerical kinematic values; two observations marked for review were retained. The worksheet preserved the inclusion/exclusion decisions but not a prespecified quantitative cutoff or a written rationale for each flag. Thus, the original operational criterion could not be fully reconstructed, and this step was treated as investigator judgment rather than a formal statistical-outlier procedure. Participants were included only if at least three valid observations were available for every combination of condition and propulsion cycle. Of the 22 participants who underwent testing, eight did not meet this criterion, leaving 14 participants for the final analysis. Among these 14 participants, 416 propulsion-cycle observations were initially identified, four fewer than the planned maximum of 420 because one three-cycle trial and the first cycle of another trial were unavailable in the archived analysis data. After the 15 exclusions described above, 401 valid propulsion-cycle observations remained. For each included participant, all available valid observations (three to five per condition and propulsion cycle) were averaged within each condition and propulsion cycle before repeated-measures analysis.

### 2.5. Statistical Analysis

Wheelchair propulsion velocity, velocity increment, and kinematic parameters were analyzed using two-way repeated-measures analyses of variance (ANOVAs). Cervical fixation condition was entered as a two-level within-subject factor, and measurement point (five levels), measurement interval (five levels), or propulsion cycle (three levels), as appropriate, was entered as the second within-subject factor. Mauchly’s test was used to assess sphericity. When sphericity was violated, Greenhouse–Geisser-corrected degrees of freedom and *p*-values were reported. Bonferroni-adjusted pairwise comparisons were used for post hoc analyses. Partial eta squared (ηp^2^) was reported as the effect size, and 95% confidence intervals were calculated for principal between-condition mean differences. Normality of within-subject difference scores was examined using Shapiro–Wilk tests; several cycle-related contrasts showed departures from normality, and these results were therefore interpreted cautiously. Statistical significance was set at *p* < 0.05. Analyses were conducted using IBM SPSS Statistics version 32.0 (IBM Corp., Armonk, NY, USA).

## 3. Results

The results for joint angles are presented in Table 2, and those for angular acceleration are presented in Table 3.

### 3.1. Wheelchair Propulsion Velocity and Its Increment

**Figure 7:**
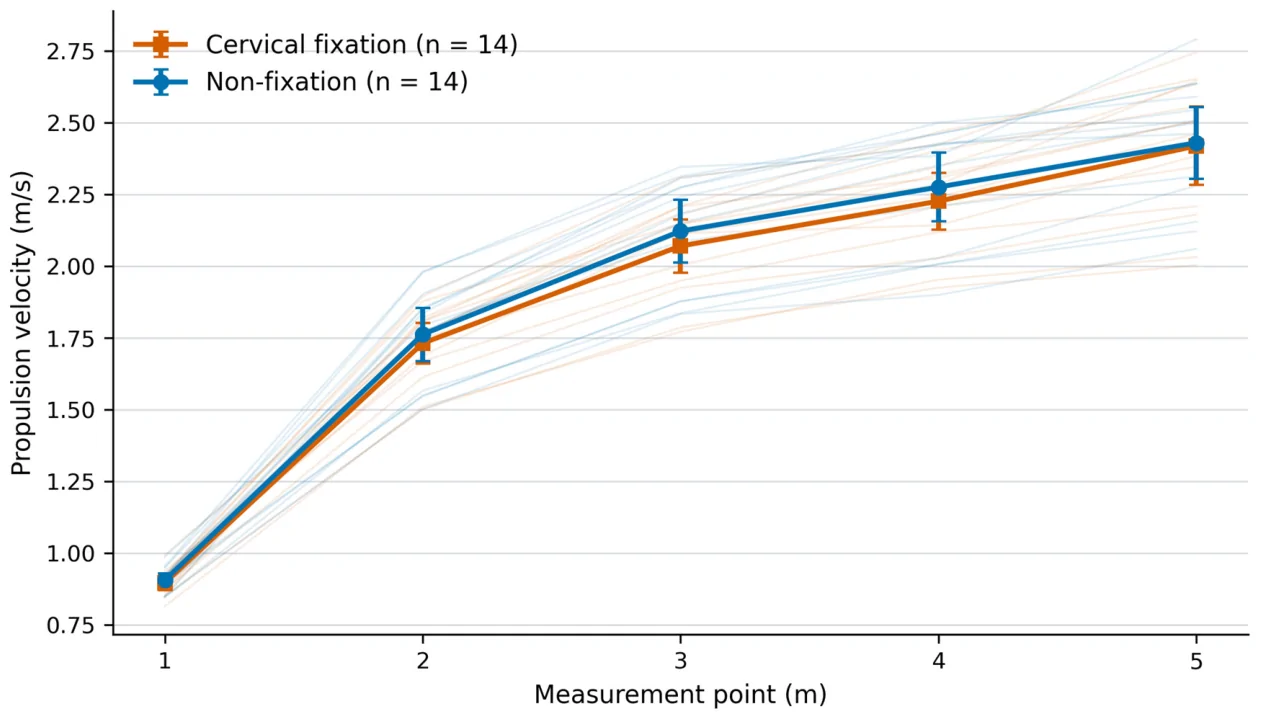
Wheelchair propulsion velocity. Bold lines and markers show condition means; error bars show 95% confidence intervals. Light lines show individual participant trajectories (*n* = 14). The main effect of measurement point was significant (Greenhouse–Geisser-corrected *p* < 0.001), whereas the main effect of condition and the condition-by-point interaction were not significant.

For propulsion velocity, the main effect of measurement point was significant after Greenhouse–Geisser correction (F(1.212, 15.754) = 712.595, *p* < 0.001, ηp^2^ = 0.982). The main effect of cervical fixation condition (F(1, 13) = 4.037, *p* = 0.066, ηp^2^ = 0.237) and the condition-by-point interaction (F(4, 52) = 1.350, *p* = 0.264, ηp^2^ = 0.094) were not significant. The overall mean difference between the non-fixation and fixation conditions was 0.030 m/s (95% CI, −0.002 to 0.063 m/s). Propulsion velocity increased progressively with measurement point in both conditions, but the present data did not demonstrate a statistically significant effect of cervical fixation on propulsion velocity (Figure 7).

**Figure 8:**
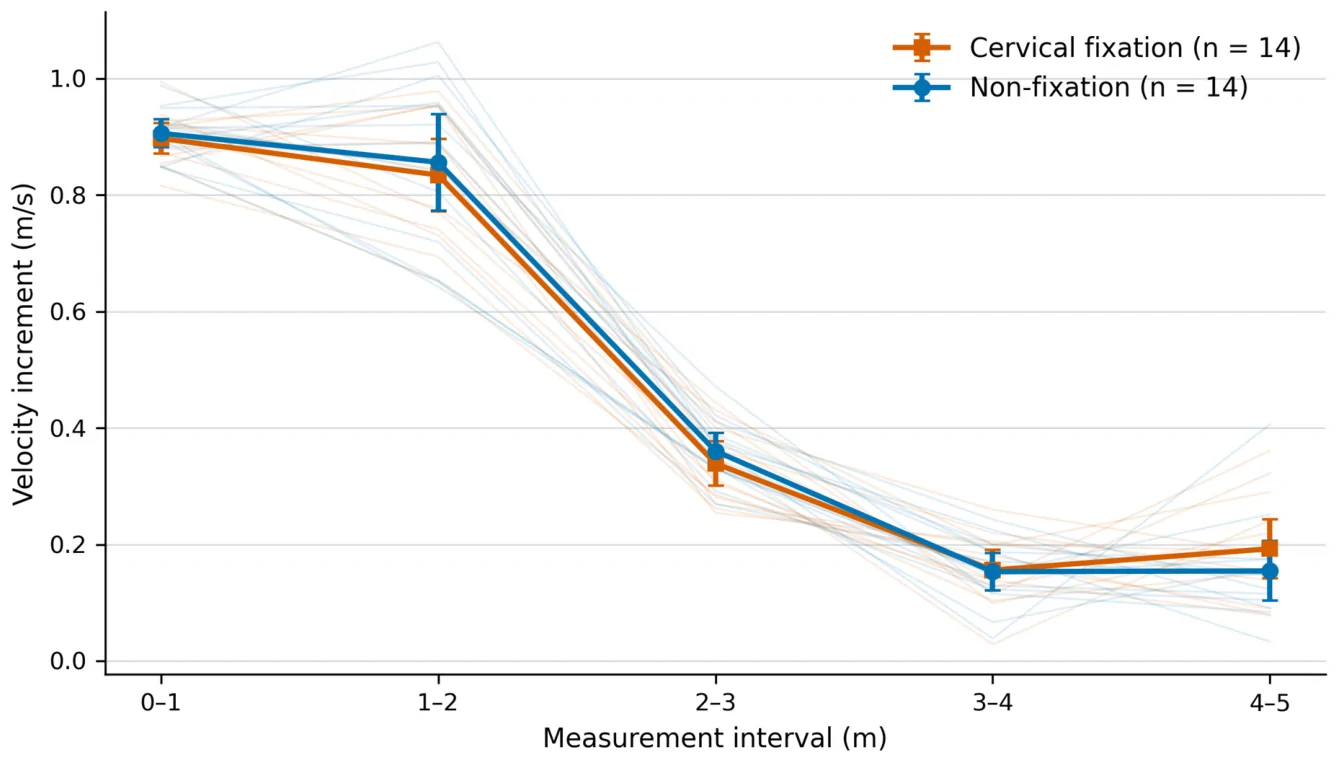
Wheelchair velocity increment. Bold lines and markers show condition means; error bars show 95% confidence intervals. Light lines show individual participant trajectories (*n* = 14). The main effect of measurement interval was significant (Greenhouse–Geisser-corrected *p* < 0.001), whereas the main effect of condition and the condition-by-interval interaction were not significant. Bonferroni-adjusted comparisons showed no significant difference between 0–1 and 1–2 m or between 3–4 and 4–5 m.

For velocity increment, the main effect of measurement interval was significant after Greenhouse–Geisser correction (F(2.325, 30.230) = 478.172, *p* < 0.001, ηp^2^ = 0.974). The main effect of condition (F(1, 13) = 0.189, *p* = 0.671, ηp^2^ = 0.014) and the condition-by-interval interaction (F(4, 52) = 1.205, *p* = 0.320, ηp^2^ = 0.085) were not significant. The 0–1 m and 1–2 m increments did not differ significantly from each other, but both were greater than the increments in the subsequent intervals. The 3–4 m and 4–5 m increments also did not differ significantly. Thus, the increase in propulsion velocity was concentrated within the initial 2 m and declined thereafter (Figure 8).

### 3.2. Upper-Limb Joint Angles

Significant main effects of cervical fixation condition were observed for minimum upper-arm segment angle (F(1, 13) = 6.739, *p* = 0.022, ηp^2^ = 0.341), upper-arm segment excursion (F(1, 13) = 5.867, *p* = 0.031, ηp^2^ = 0.311), maximum elbow angle (F(1, 13) = 6.121, *p* = 0.028, ηp^2^ = 0.320), and elbow range of motion (F(1, 13) = 16.785, *p* = 0.001, ηp^2^ = 0.564). Relative to non-fixation, fixation was associated with a smaller minimum upper-arm segment angle (mean difference, −4.46°; 95% CI, −8.16° to −0.75°), greater upper-arm segment excursion (4.05°; 95% CI, 0.44° to 7.67°), greater maximum elbow angle (4.68°; 95% CI, 0.59° to 8.76°), and greater elbow range of motion (5.89°; 95% CI, 2.78° to 8.99°).

A significant condition-by-cycle interaction was observed for minimum elbow angle (F(2, 26) = 4.600, *p* = 0.019, ηp^2^ = 0.261). In the first propulsion cycle, the fixation condition showed a smaller minimum elbow angle than the non-fixation condition (*p* < 0.001); no condition difference was detected in the second or third cycle.

Significant cycle effects were observed for maximum upper-arm segment angle (F(1.050, 13.656) = 24.074, *p* < 0.001, ηp^2^ = 0.649), minimum upper-arm segment angle (F(1.134, 14.742) = 24.323, *p* < 0.001, ηp^2^ = 0.652), maximum elbow angle (F(1.291, 16.782) = 26.183, *p* < 0.001, ηp^2^ = 0.668), and minimum elbow angle (F(1.407, 18.296) = 112.244, *p* < 0.001, ηp^2^ = 0.896). Elbow angles generally increased from the first to the third cycle, whereas upper-arm segment angles decreased. Neither upper-arm segment excursion nor elbow range of motion showed a significant cycle effect.

### 3.3. Upper-Limb Joint Angular Acceleration

For angular acceleration, a significant main effect of propulsion cycle was observed only for the minimum upper-arm segment angular acceleration in the flexion direction (F(1.103, 14.336) = 29.163, *p* < 0.001, ηp^2^ = 0.692). Bonferroni-adjusted comparisons showed progressively more negative values from the first to the third cycle. No significant condition effects or condition-by-cycle interactions were detected for the angular-acceleration variables.

## 4. Discussion

The present study examined wheelchair propulsion velocity and upper-limb kinematics under cervical fixation and non-fixation conditions in healthy individuals. Propulsion velocity and velocity increment changed substantially across measurement points or intervals, but neither outcome showed a statistically significant condition effect or interaction. In contrast, cervical fixation was associated with differences in upper-arm segment and elbow kinematics, including greater upper-arm segment excursion and elbow range of motion. The first propulsion cycle also showed a fixation-related decrease in minimum elbow angle. These findings indicate that the fixation condition was associated with altered upper-limb kinematics, although a corresponding change in propulsion velocity was not statistically demonstrated.

The elbow range-of-motion values observed in this study were broadly similar to those reported by Boninger et al. during manual wheelchair propulsion at two speeds [16]. However, the variable originally described as shoulder angle was defined relative to the horizontal axis rather than to the trunk; it is therefore more accurately termed the upper-arm segment angle and is not directly equivalent to an anatomical shoulder joint angle. Direct comparison with previously reported shoulder joint ranges of motion is consequently limited. The time-series angular-acceleration waveforms nevertheless showed characteristic propulsion-related patterns, including a bimodal elbow waveform and coordinated flexion–extension changes of the upper-arm segment.

Mean propulsion velocity was descriptively lower under fixation at most measurement points, but the condition effect did not reach statistical significance and the 95% confidence interval for the overall condition difference included zero. Accordingly, the present results do not establish that cervical fixation reduces propulsion velocity. The significant kinematic differences suggest that participants adopted a different upper-limb movement strategy under fixation without a statistically demonstrable change in propulsion performance. Potential explanations involving altered head–neck–trunk coordination, force generation, or elbow-flexor recruitment remain hypotheses because cervical and trunk kinematics, propulsive force, and muscle activity were not measured.

The findings may provide a basis for future research in wheelchair rugby. Because the present participants were healthy individuals and cervical, head, and trunk kinematics were not directly quantified, the results do not directly support changes in coaching or classification practice. Studies involving wheelchair-rugby athletes and individuals with tetraplegia are required to determine whether the observed associations are relevant to sport-specific performance or classification.

Several limitations should be acknowledged. First, only the left upper limb was analyzed, and the analysis was limited to the sagittal plane; bilateral coordination and frontal-plane motion could not be evaluated. Second, cervical angle, cervical range of motion, and head–trunk coordination were not quantified. The collar may have altered the head–neck–trunk configuration rather than solely restricting cervical motion. Third, propulsion velocity was calculated by a single unblinded examiner using visual frame counting. Although videos were reviewed multiple times, intra-rater and inter-rater reliability were not formally quantified. At 30 Hz, the temporal resolution was approximately 0.033 s, corresponding to an estimated one-frame velocity difference of approximately 0.03–0.05 m/s across the observed speeds. Fourth, numerical differentiation may amplify small errors in two-dimensional pose estimation, and the 30 Hz sampling rate may limit the precision of peak angular-acceleration estimates. Although Gaussian smoothing was applied before differentiation, these values should be interpreted cautiously. In addition, the archived materials did not retain the exact YOLOv7 model weights and implementation version, confidence threshold, exact coordinate-position filtering thresholds, interpolation records, or original execution environment. The original preprocessing procedure therefore could not be fully reconstructed, and no study-specific manual annotation or positional or angular accuracy validation was available. Fifth, the archived worksheet identified 15 excluded observations and two observations marked for review that were retained, but it did not preserve a prespecified quantitative cutoff or a written rationale for each decision. Because the same investigator who collected the data performed the review, subjectivity cannot be excluded. Sixth, a single fixed wheelchair configuration was used without participant-specific adjustment, and tire pressure was not remeasured before every participant. These procedural features may have introduced between-participant variability, although the configuration was kept constant between conditions within each participant. Seventh, kinetic and electromyographic measurements were not obtained, so the mechanical and neuromuscular mechanisms remain unclear. Eighth, the sample was small (*n* = 14), several cycle-related difference-score distributions departed from normality, and the 95% confidence interval for the principal propulsion-velocity condition difference included zero; the precision and generalizability of the estimates are therefore limited. Finally, all participants were healthy young men without habitual wheelchair use or wheelchair-sport experience, rather than wheelchair-rugby athletes or individuals with tetraplegia, and the findings should not be extrapolated directly to those populations.

## 5. Conclusions

This study compared wheelchair propulsion under cervical fixation and non-fixation conditions in healthy individuals. Cervical fixation was associated with differences in upper-arm segment and elbow kinematics, including greater segment excursion and elbow range of motion. However, no statistically significant condition effect was demonstrated for propulsion velocity or velocity increment. Because cervical kinematics, propulsive force, and muscle activity were not measured and the sample consisted of 14 healthy young men, these findings should be interpreted as preliminary biomechanical evidence. Further studies in wheelchair users and athletes are required to determine whether fixation-related kinematic changes influence sport-specific propulsion performance.

## Figures and Tables

**Table 1:** Participant characteristics.

Characteristic		
Age (years)	20.7 (0.5)	
Height (cm)	175.4 (4.6)	
Weight (kg)	73.9 (10.7)	
Sitting height (cm)	92.8 (2.5)	
	Right	Left
Upper arm length (cm)	30.7 (1.6)	30.5 (1.5)
Forearm length (cm)	27.0 (1.7)	27.0 (1.6)
Hand length (cm)	19.8 (1.2)	19.6 (1.0)
Grip strength (kg)	48.2 (5.7)	46.3 (6.3)

Values are presented as mean (SD).

**Table 2:** Joint angles during wheelchair propulsion.

							Two-Way ANOVA		
	1st Cycle		2nd Cycle		3rd Cycle		*p*-Value		
	Cervical Fixation	No Fixation	Cervical Fixation	No Fixation	Cervical Fixation	No Fixation	Condition	Propulsion Cycle	Interaction
Max upper-arm segment angle (°)	149 ± 6 ^de^	149 ± 7 ^e^	145 ± 8 ^ce^	146 ± 8 ^e^	139 ± 9 ^cd^	140 ± 11 ^cd^	0.631	<0.001	0.534
Min upper-arm segment angle (°)	95 ± 11 ^de^	102 ± 20 ^e^	89 ± 11 ^ce^	91 ± 11 ^e^	81 ± 10 ^cd^	85 ± 12 ^cd^	0.022	<0.001	0.133
Upper-arm segment excursion (°)	54 ± 12	47 ± 21	56 ± 14	55 ± 14	58 ± 13	55 ± 14	0.031	0.145	0.175
Max elbow angle (°)	114 ± 12 ^de^	106 ± 20 ^e^	120 ± 11 ^ce^	118 ± 13 ^e^	130 ± 7 ^cd^	126 ± 10 ^cd^	0.028	<0.001	0.263
Min elbow angle (°)	55 ± 7 ^bde^	58 ± 8 ^de^	62 ± 7 ^ce^	63 ± 8 ^ce^	71 ± 9 ^cd^	71 ± 10 ^cd^	0.149	<0.001	0.019
Elbow ROM (°)	58 ± 15	48 ± 21	58 ± 14	56 ± 16	59 ± 13	54 ± 14	0.001	0.313	0.161

Values are presented as mean ± standard deviation. *p*-values were determined using two-way repeated-measures ANOVA. For effects involving propulsion cycle, Greenhouse–Geisser-corrected *p*-values are reported when Mauchly’s test indicated a violation of sphericity. Superscript b indicates a significant simple condition effect within the corresponding cycle. Superscripts c, d, and e indicate significant Bonferroni-adjusted pairwise differences from the 1st, 2nd, and 3rd propulsion cycles, respectively (adjusted *p* < 0.05).

**Table 3:** Angular acceleration during wheelchair propulsion.

							Two-Way ANOVA		
	1st Cycle		2nd Cycle		3rd Cycle		*p*-Value		
	Cervical Fixation	No-Fixation	Cervical Fixation	No-Fixation	Cervical Fixation	No-Fixation	Condition	Propulsion Cycle	Interaction
Max upper-arm segment (°/s^2^)	4293 ± 933	3818 ± 1718	4567 ± 849	4677 ± 830	4441 ± 500	4674 ± 705	0.697	0.087	0.102
Min upper-arm segment (°/s^2^)	−2697 ± 593 ^de^	−2497 ± 1049 ^de^	−3116 ± 600 ^ce^	−3199 ± 599 ^ce^	−3534 ± 496 ^cd^	−3550 ± 549 ^cd^	0.772	<0.001	0.142
Max elbow 1 (°/s^2^)	3218 ± 994	2890 ± 1451	3428 ± 830	3565 ± 849	3464 ± 687	3662 ± 695	0.981	0.064	0.073
Max elbow 2 (°/s^2^)	2651 ± 683	2759 ± 752	2928 ± 848	3051 ± 897	2870 ± 698	2962 ± 867	0.365	0.105	0.980
Min elbow (°/s^2^)	−5924 ± 1464	−4999 ± 2431	−6241 ± 1511	−6269 ± 1551	−6281 ± 1025	−6232 ± 1237	0.059	0.076	0.123

Values are presented as mean ± standard deviation. *p*-values were determined using two-way repeated-measures ANOVA. For effects involving propulsion cycle, Greenhouse–Geisser-corrected *p*-values are reported when Mauchly’s test indicated a violation of sphericity. Superscripts c, d, and e indicate significant Bonferroni-adjusted pairwise differences from the 1st, 2nd, and 3rd propulsion cycles, respectively (adjusted *p* < 0.05).

## Data Availability

The raw data supporting the conclusions of this article will be made available by the authors on request.

## Abbreviations

The following abbreviations are used in this manuscript:

BMI	body mass index
YOLO v7	You Only Look Once version 7
